# Gut Hormone Levels During Mixed Liquid Meal Test in Short Bowel Syndrome, the Possible Influence on the Intestine Adaptation

**DOI:** 10.33549/physiolres.935559

**Published:** 2025-08-01

**Authors:** Jarmila KRIZOVA, Pavel TRACHTA, Milos MRAZ, Tomas BRUTVAN, David HOSKOVEC, Petr DYTRYCH, Anna CINKAJZLOVA, Otakar PSENICKA, Martin HALUZIK

**Affiliations:** 1 3^rd^ Department of Internal Medicine, Department of endocrinology and metabolism, General University Hospital in Prague, 1^st^ Faculty of Medicine, Charles University, Prague, Czech Republic; 2 Department of Anesthesiology and Intensive Care, General University Hospital in Prague, 1st Faculty of Medicine, Charles University, Prague, Czech Republic; 3 Institute for Clinical and Experimental Medicine, Prague; 4 1^st^ Surgical Department, General University Hospital in Prague, 1^st^ Faculty of Medicine, Charles University, Prague, Czech Republic

**Keywords:** Short bowel syndrome, GLP-1, GLP-2, Mixed liquid meal test, Bowel adaptation

## Abstract

Short bowel syndrome (SBS) is an intestinal disorder characterized by reduced length of the gut most due to intestinal resection, resulting in malabsorption, malnutrition, and water and electrolyte disturbances. Intestinal adaptation is a long-term process in which GIT hormones, growth peptides, cytokines etc. are involved. These mechanisms have not been fully clarified yet. The most important seem to be GLP-2 and other gut hormones. The aim of our study was to consider the changes of the levels of selected gut hormones and parameters of glucose homeostasis during the mixed liquid meal test in one year of follow up after the gut resection. Seventeen patients with SBS type I were included into our study. The meal test and measuring of selected parameters (GLP-2, GLP_-_1, ghrelin, insulin, glucagon, GIP, amylin) were conducted after 2 weeks, 6 and 12 months from its initiation, respectively. During one year of this study patients’ nutritional status improved due to sufficient parenteral nutrition, despite no change in body weight. Hormones possibly involved in intestinal adaptation (GLP-2, GLP_-_1, ghrelin) did not differ in meal test, neither levels nor AUC. Only higher insulin and glucose levels after one year of follow-up may indicate the beginning of intestinal adaptation process and improving intestinal functions. We conclude that impaired GLP-2 secretion is probably the main reason for the limited adaptation ability in patients with SBS type I.

## Introduction

Short bowel syndrome (SBS) is a condition in which the small intestine is unable to absorb sufficient amount of nutrients and fluids, usually due to the surgical removal of a significant portion of the small intestine. However, the absorption of nutrients gradually increases after the surgery, this process is known as gut adaptation. Post-operative adaptive changes in the small intestine involve the structure of the intestinal mucosa, peristaltic activity, and the function of individual enterocytes. Structural changes occur in all layers of the intestinal wall. Increased blood flow through the intestine, causes cell proliferation, and increases the volume of the intestinal mucosa, thus enhances the absorptive surface of the small intestine [1,2]. The ileum has a higher adaptive capacity than the jejunum, making its preservation highly desirable. One reason for this is that the ileum is exposed to chyme with a significantly different composition and consistency compared to physiological conditions, receiving more substantial stimuli than the jejunum. Major changes in peristaltic rate occur in the jejunum, with peristalsis being slowed down. The deceleration of motility in the proximal sections of the digestive tract is likely induced by humoral stimuli from the ileum – known as the ileal brake [3]. Functional changes involve improving the absorption of nutrients, water, and minerals at the level of individual enterocytes. These changes are also likely influenced by humoral factors [3].

The exact mechanisms responsible for intestinal adaptation are not fully understood. Hormones, local regulatory peptides, growth factors, complex intracellular signalling cascades, cytokines, tissue factors [4, 5], and the gut microbiota [6] are certainly involved in this process. Gut hormones especially glucagon-like peptide 2 (GLP-2), glucagon-like peptide 1 (GLP-1), amylin and ghrelin very likely play the most crucial role in this process (Table 1).

For the intestinal epithelium, the most significant trophic factor is likely GLP-2. It is secreted in the ileum and the large intestine. Partial jejuno-ileal resection leads to increased GLP-2 secretion, subsequently resulting in mucosal hyperplasia. It is assumed that impaired intestinal adaptation and long-term patients’ dependence on parenteral nutrition after ileal and large intestine resection are mainly due to the lack of GLP-2 production in the body.

Even patients with ileal resection but preserved large intestine had higher levels of GLP-2 and GLP-1, indicating the importance of preserving the large intestine for intestinal motility and functional adaptation [9]. GLP-2 is currently a registered medication for SBS patients indicated to accelerate intestinal adaptation after the resection. Its administration reduces stool or stoma output, decreases thirst, and subsequently reduces the need for parenteral nutrition, improving the quality of life [18].

Similarly, GLP-1 is considered one of the mediators slowing the passage in proximal sections of the GIT [11]. However, it has more significant effect on nutrient assimilation, insulin secretion, and intestinal permeability reduction [19]. In healthy volunteers, GLP-1 release occurs after stimulation of more than 60 cm of the small intestine; it is not effective with shorter intestine lengths [19]. In a study by Madsen [20], administering GLP-1 and GLP-2 to SBS patients reduced stoma output, and losses of energy and minerals.

Amylin is a glucoregulatory hormone and a regulator of energy metabolism [13]. It is hypothesized that reduced plasma amylin levels after the bowel resection may play a role in resection-induced hyperglycemia. An experimental study demonstrated exogenous GLP-2 as an amylin secretagogue [21].

Ghrelin is supposed to be involved in adaptation to intestinal resection and may contribute to compen-satory hyperphagia, a highly desirable phenomenon after resection, speeding up the cessation of parenteral nutrition [5]. Ghrelin administration in some studies stimulates morphological intestinal adaptation [15]. Its levels in animal models peaked the first day after the resection [22], but the influence on adaptation is not clear.

Recent research focuses on ways to enhance this process; otherwise, the treatment remains primarily symptomatic. It involves the correction of the internal environment and parenteral nutrition. A residue-free form of diet and fat limitation are also essential. Symptomatic pharmacotherapy mainly includes the administration of loperamide and other medications to slow down intestinal peristalsis. Efforts to improve the absorption function of the intestine, and thus enhance the quality of life for patients after extensive bowel resection, have led to the development of drugs influencing intestinal adaptation. Currently, the injectable recombinant GLP-2 analog teduglutide is used, which reduces accelerated gastric emptying and gastric hypersecretion, increases intestinal perfusion, promotes the growth of enterocytes, increases villus height and crypt depth in the intestinal epithelium, improves intestinal barrier function, and accelerates the process of intestinal adaptation [18]. Even new GLP-2 analogues have been explored as potential treatments for SBS. Glepaglutide, a long-acting analogue, demonstrated promising results in animal models, including intestinal growth and villous hypertrophy. Its effects were observed after just one week and persisted even after six weeks of recovery, indicating that glepaglutide has both a rapid onset and a sustained impact [23]. Studies involving this peptide in humans have yielded promising conclusions; however, clinical phase 3 trials are still underway [23]. The main limitation for wider use is the cost of this treatment. In some cases, surgical enlargement of the absorption area of the small intestine or intestinal transplantation may be possible.

Nevertheless, there is still a lot unknown about the gut adaptation process. Enteral nutrition definitely provides more physiological stimulation of the gut than parenteral nutrition and can help to maintain gut hormone production and gut microbiota composition. Not just basal levels of gut hormones but even different postprandial reactivity can influence the remaining intestinal loops and may lead to the process of gut adaptation. Additionally, different nutrient absorption dynamics and postprandial nutrient levels can represent the impulse for the adaptation process. The aim of our study was to consider the changes of the levels of selected gut hormones and parameters of glucose homeostasis during the mixed liquid meal test in one year of follow-up after gut resection. According to available knowledge, this topic has not yet been published.

## Patients and Methods

### Study subjects

Seventeen patients with new onset SBS type I after jejunal resection were included into our study. The reasons for resections were tumors, post-radiation damage, intestinal injury caused during surgery, myxoma of the appendix or vascular ileus. The average remaining intestinal length was 110.7 ± 22.88 cm. The examination was carried out at the time of patient enrollment in the study (approx. 1 week after the surgery), after 2 weeks, after 6 months, and after 1 year from the enrollment. At the time of examination there were no signs of infection. All patients were dependent on parenteral nutrition that was not changed during the year; all had even regular food intake. Written informed consent was signed by each subject and the studies were approved by the Human Ethics Committee, First Faculty of Medicine and General University Hospital, Prague, Czech Republic.

### Anthropometric examination and sampling

All subjects enrolled in the study underwent anthropometric examination and their BMI was calculated.

A two-hour dynamic meal test was performed with the administration of a predefined diet (commercially produced Fresubin Vanilla 200 ml, Fresenius Kabi Deutschland GmbH, Bad Homburg, Germany) after overnight fasting with blood sampling and glycemic assessment always before the sipping administration (time 0 min) and at 5, 15, 30, 60, 90, and 120 minutes of the test. The composition of the administered diet was as follows: energy 840 kJ (200 kcal), fats 6.8 g (saturated fatty acids 0.6 g; monounsaturated fatty acids 4.4 g; polyunsaturated fatty acids 1.8 g), carbohydrates 27.6 g (sugars 7 g), proteins 7.6 g, fiber 0 g, osmolarity 660 mosmol/l.

Blood samples collected at the beginning or during the meal test were centrifuged within 30 minutes of collection for 10 minutes at 1000 × g.

Serum or plasma aliquots with K2EDTA were subsequently stored at −80 °C. Blood samples for gut hormone assays were collected into blood tubes with an inhibitor cocktail consisting of dipeptidylpeptidase 4, esterase and protease inhibitors (BD P800, Becton, Dickinson and Company, Franklin Lakes, NJ, USA). Plasma for analysis of ghrelin was modified by the addition of an inhibitor mix (protease inhibitors, dipeptidylpeptidase 4 and Defabloc SC – AEBSF) and 0.05N HCl.

### Hormonal and biochemical assays

The levels of individual gut hormones were measured using the MILLIPLEX MAP Human Metabolic Hormone Magnetic Bead Panel kit (Merck KGaA, Darmstadt, Germany). GLP-2 measurement was performed from plasma samples using a commercial ELISA kit (Merck KGaA, Darmstadt, Germany). Ghrelin measurement was performed from a blood sample collected into K2EDTA with the DPP-4 and protease inhibitors using a commercial ELISA kit (Merck KGaA, Darmstadt, Germany). Serum citrulline levels were analyzed using ion-exchange chromatography with ninhydrin detection on an automatic amino acid analyzer (INGOS s.r.o., Prague, Czech Republic). The analysis was conducted in collaboration with RNDr. MUDr. Pavel Ješina, Ph.D., at the Institute of Inherited Metabolic Disorders, General University Hospital in Prague.

Biochemical parameters (blood glucose, glycated hemoglobin – HbA_1c_, HDL cholesterol, total cholesterol, triglycerides and CRP levels) were measured and LDL cholesterol was calculated at the Department of Biochemistry, General University Hospital, Prague, Czech Republic by standard laboratory methods.

### Statistical analysis

Statistical analysis was performed, and graphs were drawn using SigmaPlot 13.0 software (SPSS Inc., Chicago, IL, USA). Results are presented as mean ± standard error of the mean (SEM). To analyze differences between individual visits, One way RM ANOVA followed by the Holm-Sidak test or One way RM ANOVA on Ranks followed by the Dunn method was used, depending on the normality of the data. Differences during meal tests were evaluated by calculating areas under the curve within individual visits, followed by One way RM ANOVA followed by the Holm-Sidak test or One way RM ANOVA on Ranks followed by the Dunn method. Additional statistical analyses were used as needed under conditions necessary for their use. The level of statistical significance was determined as p<0.05.

## Results

**Fig. 1:**
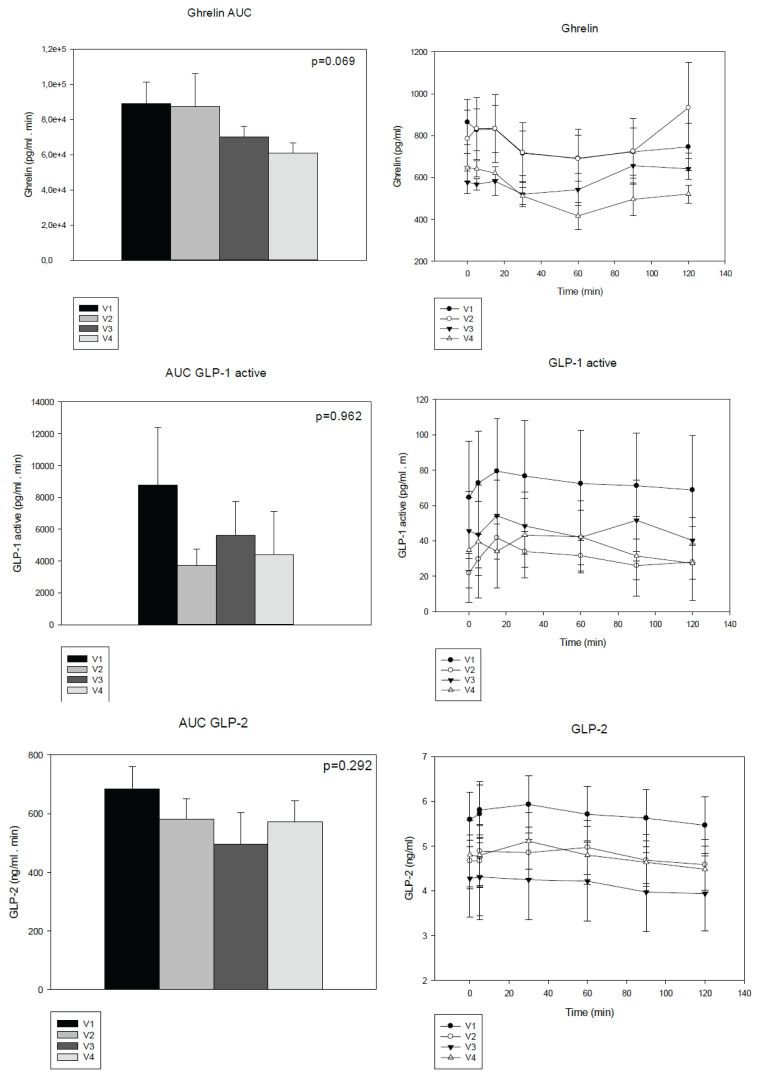
Selected hormones levels during meal test in patients after the gut resection (on the right side), and AUC (Area under the curve) during this test (on the left side). GLP-1 glucagon like peptide 1, GLP-2 glucagon like peptide 2. V1: after the surgery, V2: after 2 weeks from V1, V3: 6 months after the surgery, V4: 1 year after the surgery. Values are presented as mean ± SEM.

In all 17 patients with short bowel syndrome included in the project, a trend towards increased body weight was observed during one year of follow-up. There was a significant increase in albumin, transferrin and hemoglobin levels. Other parameters – glycaemia and lipid profile remained unchanged (Table 2). During planned check-ups, a meal test was repeatedly performed on all patients. The levels of selected hormones (ghrelin, GLP-1, GLP-2, GIP) were evaluated during the test, and for comparison, the area under the curve (AUC) during the meal test at each check-up was also assessed. Over the course of a year, there was no statistically significant change in the AUC of values during the meal test nor different dynamics of the release of the monitored substances. For ghrelin, from V3 (six months after bowel resection), there was an observable decrease in levels, but it did not reach statistical significance (p=0.069) (Fig. 1).

**Fig. 2:**
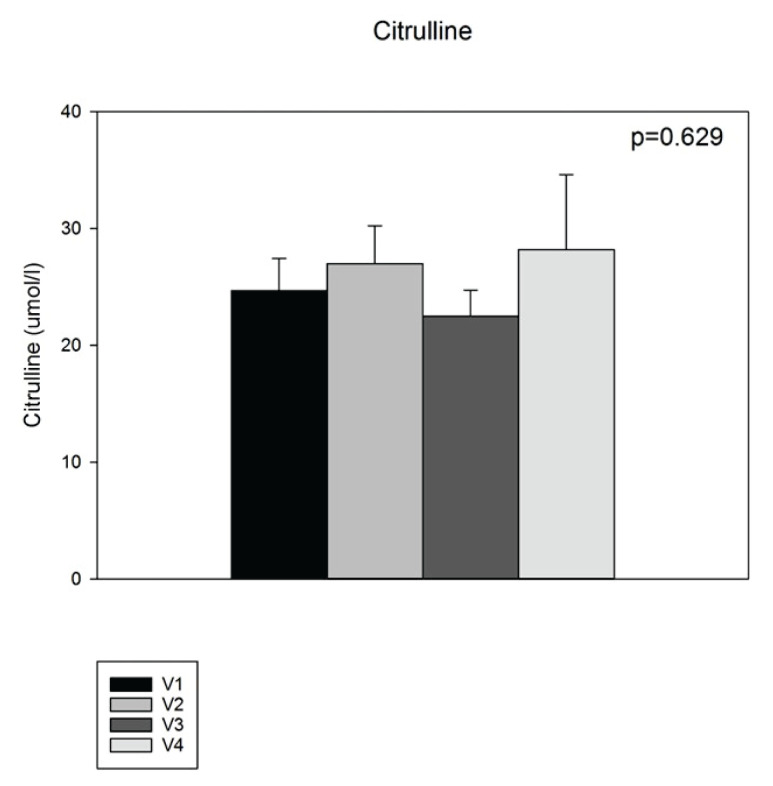
Citrullin levels in patient after the gut resection during one year of follow-up. V1: after the surgery, V2: after 2 weeks from V1, V3: 6 months after the surgery, V4: 1 year after the surgery. HC – healthy controls. Values are presented as mean ± SEM.

To evaluate the degree of intestinal adaptation we measured citrullin levels during one year and compared the levels with healthy age matched people (Fig. 2).

**Fig. 3:**
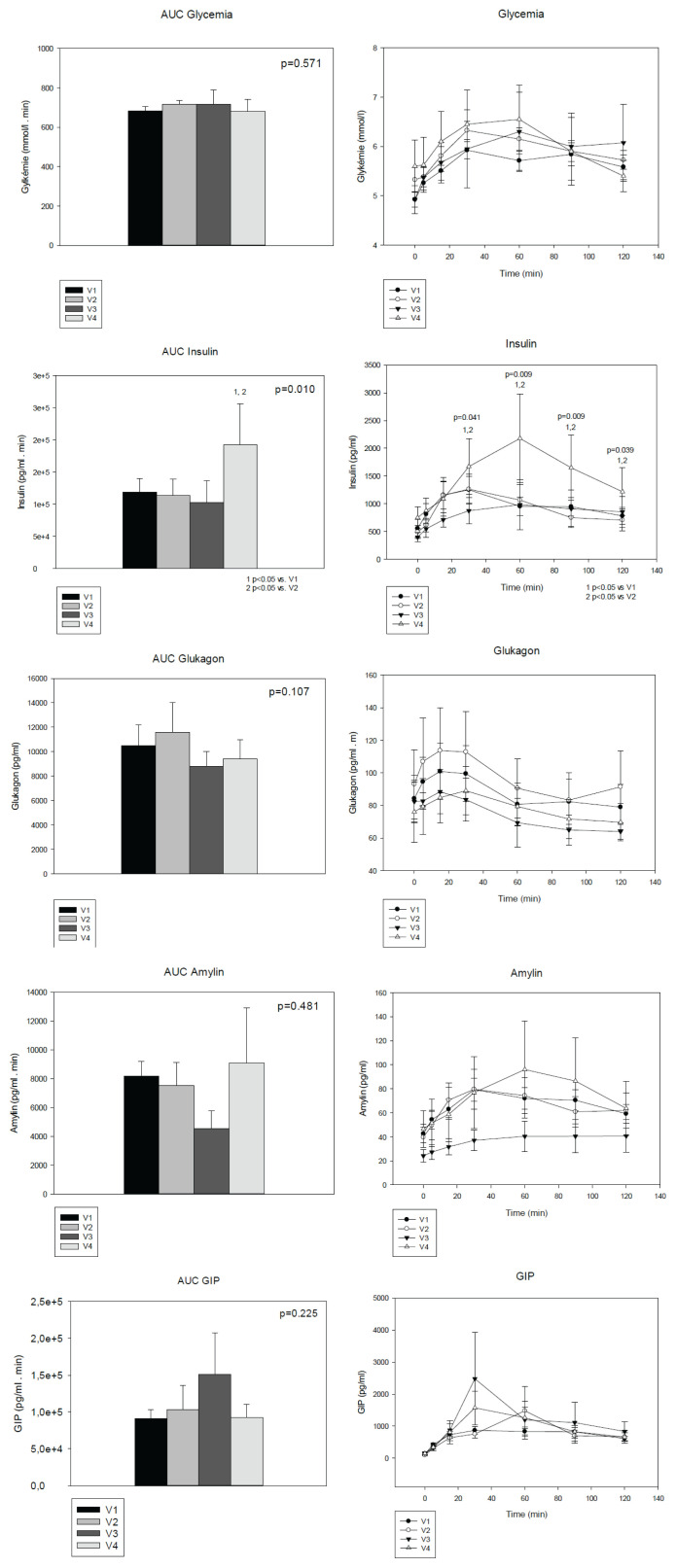
Selected parameters of glucose metabolism during meal test in patients after the gut resection (on the right side), and AUC (Area under the curve) during this test (on the left side). GIP: Gastric inhibitory peptide, V1: after the surgery, V2: after 2 weeks from V1, V3: 6 months after the surgery, V4: 1 year after the surgery. Values are presented as mean ± SEM. ^1^ p<0.05 V1 vs. V4 ^2^ p<0.05 V2 vs. V4

When monitoring changes in nutrient absorption, we measured blood glucose, insulin, glucagon, and amylin levels in patients with short bowel syndrome during a meal test. The levels of total amylin and glucagon did not differ significantly during the meal test or between individual visits, and the dynamics of the secretion of these hormones did not change due to ongoing adaptation. The fasting blood glucose values and glycated hemoglobin levels were not significantly affected during the study. When examining dynamic changes during the meal test, we observed only a tendency of blood glucose to be increased at 60-minutes visit V4. Insulin levels were elevated at 30, 60, 90 and 120 minutes of the meal test at V4 compared to V1 and V2, and the AUC of insulin levels was also higher at V4 compared to V1 and V2 (Fig. 3).

## Discussion

There is still a lot unknown about the gut adaptation process after bowel resection. It is clear that the most important role in adaptation plays GLP-2. Its secretion is definitely promoted by different chymus composition in ileum when jejunum missing [25]. And even GLP-2 analogue teduglutide effect on adaptation is enhanced by oral food intake [26]. It is very likely that other hormones and factors are involved in this process. An important part of bowel rehabilitation after resection is enteral feeding. Therefore, we hypothesized that even different reactions to meal intake can be the impulse for changed gut peptides secretion and the adaptation process. The aim of our study was to compare selected hormone levels during mixed a liquid meal test in patients with SBS type 1 in a one year follow-up. Standard meal test results were published for example in rats after sleeve gastrectomy, where a rapid rise in blood sugar was observed followed by reactive hypoglycaemia and increased GLP-1 [27], but no study in short bowel syndrome patients has been published yet.

During one year the body weight, BMI respectively of patients with short bowel syndrome increased but it did not reach statistical significance. The BMI didn’t increase significantly probably because of the small amount of probands. Sufficient parenteral nutrition was provided to all patients, their nutritional status was clinically improved. Increase of hemoglobin, albumin and transferrin levels were found after 6 and 12 months. However, transferrin levels shortly after surgery can be affected by systemic inflammation and do not reliably reflect nutritional status. All subjects were dependent on parenteral nutrition even after one year so the adaptation response was not fully developed. Lipid parameters did not differ between visits. Studies have shown that nutrient intake, particularly carbohydrates and fat, can stimulate GLP-2 secretion. The increase in GLP-2 levels in response to meal is part of the mechanism by which the gut adapts to changes in nutrient intake, promoting gut growth and nutrient absorption [28]. In our study, we measured some hormone levels involved in the adaptation process (GLP-2, GLP-1, ghrelin). No difference was found in the area under the curve (AUC) values during the meal test in the one year follow-up. The probable cause of unchanged GLP-2 and GLP-1 levels is the removal of the ileum, which is the main producer of these hormones, as repeatedly described in the literature. Ileum resection is a condition for worse adaptation ability and longer dependence on parenteral nutrition [9]. Impaired meal stimulated GLP-2 response in short bowel syndrome was an argument for GLP-2 analogue therapeutic strategy [29].

In our study ghrelin levels were not changed during the one year follow-up, although they had a tendency to decrease after 6 months from SBS initiation. Ghrelin administration in some studies stimulates morphological intestinal adaptation [15], but its influence on bowel adaptation is not clear. In some studies ghrelin’s influence on hyperphagia is disputed; the presence of the colon in continuity and contact with the nutritional flow seems to play the most important role in hyperphagia [30]. It was proven that fasting and postprandial plasma ghrelin and PYY concentrations were higher in jejuno-colo anastomosis patients and rats comparing to jejuno-ileo anastomosis subjects. These authors declared that the induction of orexigenic peptides such as ghrelin and PYY is stronger in the absence of the ileum and preserved colon, which maintains a specific environment promoting increased hunger signals [5]. This can explain our finding as all subjects had jejunostomy.

To verify the degree of ongoing adaptation, we measured citrulline levels at each check-up. This amino acid is not contained in the diet; it is only produced in the intestine and its levels are supposed to correspond with the volume of the intestinal mucosa. In our study, the citrulline levels did not differ even a year after the initial post-bowel resection state. This can indicate that the adaptation process did not occur and there was no increase in the mass of enterocytes. It is known that adaptation is a long-term process, especially in SBS type I with missing ileum, and longer follow up would bring significant results. There are publications that declare the same results and conclude that citrulline seems to be a not valid biomarker of bowel length or bowel absorptive function in short bowel syndrome patients [31].

Together with parameters possibly involved in intestinal adaptation glucose absorption and hormones connected in glucose metabolism were studied in our one-year follow-up. Monitoring blood glucose levels throughout the meal test revealed minor differences. Blood glucose showed a transient tendency to be increased in 60 minute of the test after 12 months of follow-up. It may somehow reflect improving enterocyte function. However unchanged glycated hemoglobin levels indicate not significant differences in average blood glucose levels. Insulin levels expressed as AUC were higher after one year of follow-up compared to levels immediately after and 2 weeks post-surgery. Also, insulin levels during the meal test were elevated at 30, 60, 90 and 120 minutes of the meal test at V4 compared to V1 and V2, while the levels of glucagon and amylin did not differ between the individual check-ups. Levels of incretins (GIP, GLP-1) remained statistically unchanged. Therefore, higher insulin levels are very likely in response to higher glucose levels not induced by incretin hormones.

GIP together with GLP-1 as incretin hormones amplify insulin production after orally taken glucose. Their secretion is very rapid after gastric emptying [32]. GIP seems to be more important; the action of GLP-1 is mediated by inhibition of glucagon secretion. GIP and GLP-1 are produced by endocrine cells in the duodenum and jejunum but also in the ileum and the colon.

Production of GIP in short bowel syndrome has not been studied yet. Surprisingly its levels in our study did not differ in the meal test during the one year follow up; only non-statistically higher levels in 30th minute of the meal test were found after 6 and 12 months. The stimulation of the upper parts of the intestine by glucose leads to a rapid increase of GIP production; therefore, our finding is quite surprising. We may hypothesize that the upper GIT transit is fast due to the missing ileal break or that some other products block incretin effects in short bowel syndrome patients.

In Lai study [21] it was presented that reduced plasma amylin after bowel resection may play a role in resection-induced hyperglycemia.

The authors hypothesized that insulin is co-secreted with amylin and follows a similar pattern. In our study amylin levels were not changed during one year, higher insulin concentrations were not dependent on amylin levels. This finding can support our hypothesis that higher insulin levels are the result of faster glucose absorption as the first signal of intestinal adaptation initialization.

Our observations support the hypothesis that gut adaptation in our patients with SBS type I after one year had not begun yet. Only a transient tendency of higher glycemias during the meal test may reflect slightly improving enterocyte function. This finding is also supported by the non-significant change in BMI and the ongoing dependence on parenteral nutrition. Our study is partially limited by the relatively low number of participants. The meal test was not conducted at later time points because many patients underwent surgical restoration of intestinal continuity and other patients died from related complications.

## Conclusions

Bowel adaptation is an important process that allows the gut to adjust to changes in nutrient intake and optimize nutrient and water absorption. Gut hormones play a key role in this process by promoting intestinal growth and enhancing nutrient absorption. Impaired GLP-2 secretion is probably the main reason for limiting adaptation ability in patients with ileal resection and this was supported even by our results. Adaptation as a long-term process in our study had not started one year after the bowel resection. Understanding the influence of bowel adaptation on gut hormone levels can provide insights into the mechanisms underlying this process and may lead to the development of novel therapies for malabsorption disorders such as short bowel syndrome.

## Figures and Tables

**Table 1:** Overview of the physiology and main functions of the monitored proteins.

Protein	Site of production	The GIT source	Action	References
*GLP-2*	Ileum Large intestine	Endocrine L cells	Increases the jejunum and ileum mass and mucosa volume Enhances crypt proliferation Reduces enterocyte apoptosis Increases the expression of mucosal digestive enzymes Supports the absorptive capacity of the intestine Enhances the expression of transport mechanisms in the intestine	[7, 8, 9, 10]
*GLP-1*	Ileum	Endocrine L cells	Slows the passage in proximal sections of the GIT Stimulates insulin secretion and inhibits glucagon secretion Controls islet proliferation Reduces intestinal permeability, motility and secretion Regulates appetite and food intake	[8, 11, 12]
*Amylin*	Pancreas	β cells	Slows gastric emptying Suppress glucagon secretion Initiates an anorectic postprandial signal Regulates energy metabolism Regulates glucose metabolism	[13, 14]
*Ghrelin*	Predominantly secreted by stomach Small intestine Pancreas Brain	Oxyntic cells/gastric fundus Pyloric gland cells Upper small intestinal cells	Affects secretion of gastric acid, gastric motility and pancreatic protein output Regulates energy metabolism, food intake, adiposity, body weight and temperature Raising blood glucose during starvation Promotes growth hormone secretion	[5, 15, 16, 17]

**Table 2:** Biochemical parameters during one year follow-up after the short bowel onset.

	V1	V2	V3	V4
*Age (years)*	64.4 ± 3.1			
*BMI (kg/m* *2* *)*	22.87 ± 4.87	25.65 ± 5.26	25.05 ± 1.63	28.10 ± 4.26
*Total protein (g/l)*	61.04 ± 9.11	69.37 ± 6.38^1^	73.85 ± 8.06	72.46 ± 7.98
*Albumin (g/l)*	26.98 ± 4.31	31.72 ± 7.38	33.55 ± 8.38	38.94 ± 3.63^1^
*Prealbumin (g/l)*	0.221 ± 0.080	0.255 ± 0.074	0.245 ± 0.062	0.264 ± 0.063
*Transferrin (g/l)*	1.892 ± 0.634	2.184 ± 0.523	2.398 ± 0.718	2.572 ± 0.625^1^
*Glycaemia (mmol/l)*	5.281 ± 0.899	5.738 ± 0.777	5.750 ± 1.660	5.380 ± 0.785
*HbA1c (mmol/mol)*	36.92 ± 6.69	43.00 ± 5.66	36.33 ± 2.31	33.20 ± 2.78
*Total cholesterol (mmol/l)*	3.664 ± 0.611	4.027 ± 0.885	2.825 ± 0.591	4.114 ± 0.613
*Triglycerides (mmol/l)*	1.734 ± 1.083	2.083 ± 0.590	1.783 ± 0.233	1.623 ± 0.644
*HDL cholesterol (mmol/l)*	0.981 ± 0.499	0.991 ± 0.366	0.890 ± 0.483	1.362 ± 0.773
*LDL cholesterol (mmol/l)*	1.976 ± 0.407	2.083 ± 0.590	1.783 ± 0.233	1.623 ± 0.644
*Hemoglobin (g/l)*	95.53 ± 7.54	107.078 ± 13.77^1^	110.67 ± 9.82	119.00 ± 17.80^1^

V1: after the surgery, V2: after 2 weeks from V1, V3: 6 months after the surgery, V4: 1 year after the surgery. Values are presented as mean ± SEM.

1 p<0.05 vs. V1;

2 p<0.05 vs. V2
